# Bones' Adaptive Response to Mechanical Loading Is Essentially Linear Between the Low Strains Associated With Disuse and the High Strains Associated With the Lamellar/Woven Bone Transition

**DOI:** 10.1002/jbmr.1599

**Published:** 2012-03-19

**Authors:** Toshihiro Sugiyama, Lee B Meakin, William J Browne, Gabriel L Galea, Joanna S Price, Lance E Lanyon

**Affiliations:** 1 School of Veterinary Sciences, University of BristolBristol, UK; 2 Centre for Multilevel Modelling, University of BristolBristol, UK

**Keywords:** BONE LOADING, STRAIN-RELATED ADAPTATION, MECHANOSTAT, LAZY ZONE, MINIMUM EFFECTIVE STRAIN

## Abstract

There is a widely held view that the relationship between mechanical loading history and adult bone mass/strength includes an adapted state or “lazy zone” where the bone mass/strength remains constant over a wide range of strain magnitudes. Evidence to support this theory is circumstantial. We investigated the possibility that the “lazy zone” is an artifact and that, across the range of normal strain experience, features of bone architecture associated with strength are linearly related in size to their strain experience. Skeletally mature female C57BL/6 mice were right sciatic neurectomized to minimize natural loading in their right tibiae. From the fifth day, these tibiae were subjected to a single period of external axial loading (40, 10-second rest interrupted cycles) on alternate days for 2 weeks, with a peak dynamic load magnitude ranging from 0 to 14 N (peak strain magnitude: 0–5000 µε) and a constant loading rate of 500 N/s (maximum strain rate: 75,000 µε/s). The left tibiae were used as internal controls. Multilevel regression analyses suggest no evidence of any discontinuity in the progression of the relationships between peak dynamic load and three-dimensional measures of bone mass/strength in both cortical and cancellous regions. These are essentially linear between the low-peak locomotor strains associated with disuse (∼300 µε) and the high-peak strains derived from artificial loading and associated with the lamellar/woven bone transition (∼5000 µε). The strain:response relationship and minimum effective strain are site-specific, probably related to differences in the mismatch in strain distribution between normal and artificial loading at the locations investigated. © 2012 American Society for Bone and Mineral Research.

## Introduction

Mechanical loading is the major functional influence on bone architecture. The purpose of this adaptive relationship is presumed to be the establishment and subsequent maintenance of bony structures that are adequate to withstand the repeated loads of everyday activity without fracture or an accumulation of microdamage that cannot be repaired by acceptable levels of remodeling. Remodeling associated with turnover, internal repair, and changes in architecture involves coordinated control of bone formation and resorption. A bone's strain experience as a result of functional loading acts as a direct and/or indirect stimulus for these processes and, in turn, is one of their important outcomes.^1^, ^2^

**Fig. 1:**
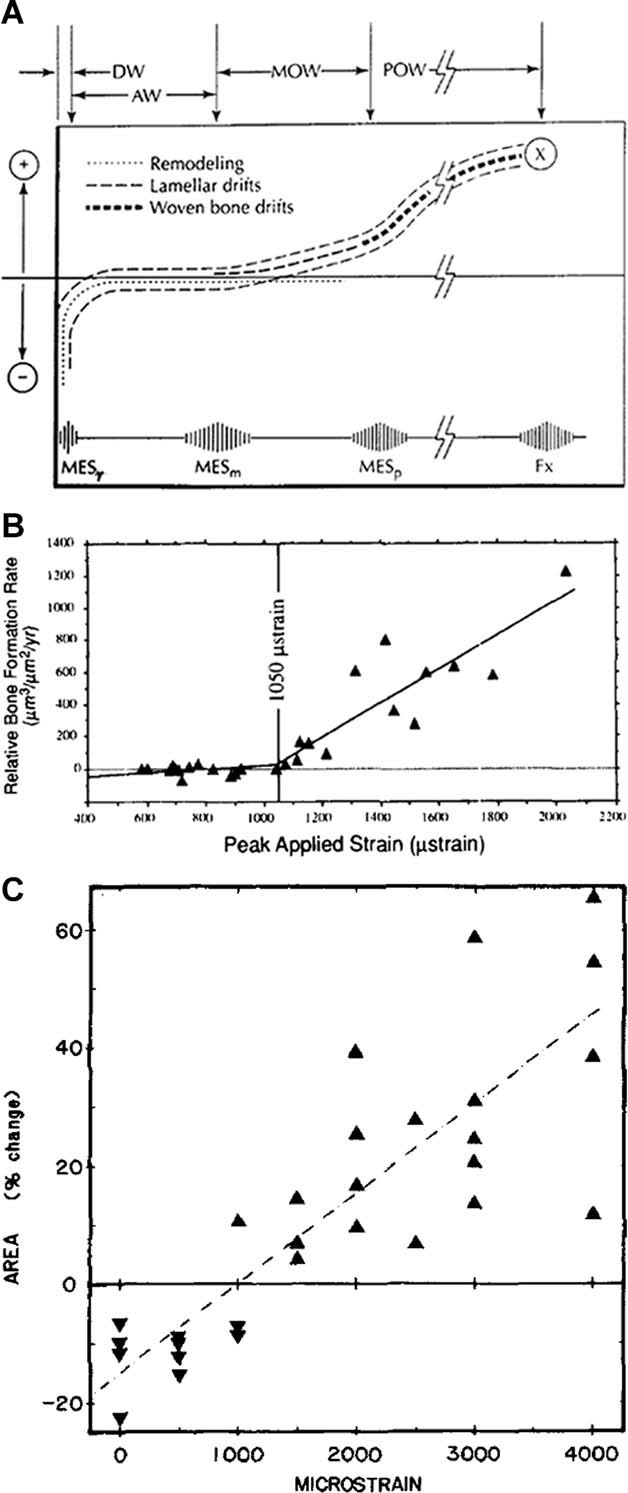
Adult bone's adaptive response to peak strain in vivo. (*A*) Relationship between peak strain (*x* axis) and bone strength (*y* axis), hypothesized by Frost,^4^ includes an unresponsive “lazy zone” (adapted window [AW]) where bone strength remains constant over a wide range of strain magnitudes. Adapted from Ref. 4. (*B*) Experimental data in “intact” cortical bone receiving natural loading, reported by Turner and colleagues,^10^ appears to support the “lazy zone” in the relationship between externally applied peak strain and the resulting change in bone formation. Adapted from Ref. 10. (*C*) Experimental data in “isolated” cortical bone protected from natural loading, reported by Rubin and Lanyon,^6^ does not support the “lazy zone” in the relationship between externally applied peak strain and the resulting change in bone area. Adapted from Ref. 6.

Harold Frost^3^ was the first to articulate the importance of functional bone strain as a controlling stimulus for bone architecture, a relationship that has come to be known as the mechanostat. The relationship between the inputs and outputs of the mechanostat has received many theoretical analyses. In adults, one hypothesis^4^ that has received widespread acceptance is that there is an adapted state called the “lazy zone” where bone mass/strength is unresponsive to a wide range of normal loading with decreases or increases in the mass/strength occurring only at the extremes of loading such as disuse or vigorous activity (Fig. 1*A*). An alternative hypothesis^5^ is that bones have a genetically determined minimum mass and that normality of bone structure results from a progressive, essentially linear, response to the strain-related stimulus they receive from exposure to functional load-bearing. This strain-related stimulus is a function of the strain waveform, such as peak strain^6^ and strain rate,^7^ and the mismatch in strain distribution from that inherent in the genetic blueprint.^8^ The “lazy zone” hypothesis was developed as an outcome of computer analysis relating measures of bone architecture to cumulative daily activity patterns in humans.^9^ It received some support from animal studies in which artificial loading and natural loading coexisted in intact bones^10^ because these studies showed no adaptive changes in bone formation below a certain level of peak strain derived from the artificial load (Fig. 1*B*). The linear hypothesis has experimental support from an in vivo loading study using surgically isolated bone segments^6^ that were protected from natural loading (Fig. 1*C*). The obvious difference between the experimental basis for these two hypotheses is the absence^6^ or presence^10^ of loading associated with normal activity.

The control processes for these two hypotheses would involve different mechanisms. In his analogy of the thermostat, Frost envisaged that normality of bone architecture was a predetermined state and that bone mass/strength was adjusted downward (by net resorption) or upward (by net formation) only when peak strains were below or above two different set points—the minimum effective strains (MESs)—that mark the lower and upper limits of the “lazy zone.”^3^, ^4^ This theory contrasts with the suggestion that bones' genetically determined state is the minimum mass associated with disuse and that normality of bone architecture results from a progressive strain-related stimulus for adaptive change within this template to achieve all the features upon which load-bearing competence depends.^1^, ^5^ In Frost's interpretation,^3^, ^4^ adaptation occurs in opposite directions at either extreme of loading, such as disuse or vigorous activity, with different control processes for bone loss and gain; the “lazy zone” is the tolerance between the two MESs (Fig. 1*A*). In Lanyon's hypothesis,^1^, ^5^ adaptation is a continuous, essentially linear, controlled process in which bone mass is a variable established and maintained as an individual achievement at each site as a result of a sustained strain-related net osteogenic stimulus opposing a (probably hormonally mediated) net drive toward resorption. Removal or reduction of the strain-related stimulus naturally results in bone loss.

Relating these two concepts of strain-related control to the natural situation in vivo is not straightforward. The “lazy zone” theory may explain why in young adult athletes significant enhancement of hip structure cannot be achieved by repetitive, lower-impact exercise such as endurance running,^11^ although this could be explained by early saturation of the strain-related stimulus.^8^ Analysis attempting to describe the natural situation mathematically by defining bones' daily strain stimulus in terms of accumulated aggregate of loading history^12^ also suggests that there is an unresponsive “lazy zone.” Similarly, animal studies in which bones receiving normal functional loading are additionally subjected to artificial loads^10^ appear to support the “lazy zone” by showing osteogenic responses only above certain levels of peak strain (Fig. 1*B*). However, because these latter studies provide a strain-related stimulus requiring adaptive change in bone structure only when applied loads engender strains higher than (or radically different in distribution from) those already being experienced, they cannot distinguish between an unresponsive zone and a zone which has already responded to the strains associated with normal activity.

We designed the present experiment to establish the nature of the adaptive response to mechanical loading in bone architecture from a situation of low strains associated with disuse, through the normal locomotor strain range, to the zone of the highest physiological strains. In this experiment, one hind limb in groups of skeletally mature mice was sciatic neurectomized to minimize stimulation from natural loading and the tibia in that limb was subjected to short periods of external axial loading sufficient in each group to engender different peak strains^6^ while keeping maximum strain rate^7^ and strain distribution^8^ constant. High-resolution micro–computed tomography (µCT) was used to quantify variables of three-dimensional cortical and trabecular bone structure at precisely comparable sites of the loaded and contralateral control limbs.

## Subjects and Methods

### Animals

The mouse has become the animal of choice for investigating bones' adaptive responses to loading,^13^ and the C57BL/6 strain has been extensively used as the background of genetically modified animals in the field of bone research. In the present study, virgin female C57BL/6 mice were purchased from Charles River Laboratories, Inc. (Margate, UK) at 16 weeks of age, and housed in cages (*n* = 6 per cage) with free access to water and a maintenance diet containing 0.75% calcium (EURodent Diet 22%; PMI Nutrition International, LLC, Brentwood, MO, USA) in a 12-hour light/dark cycle, with room temperature at 21 ± 2°C. All procedures complied with the UK Animals (Scientific Procedures) Act 1986 and were reviewed and approved by the ethics committee of the University of Bristol (Bristol, UK).

### Experimental design

**Fig. 2:**
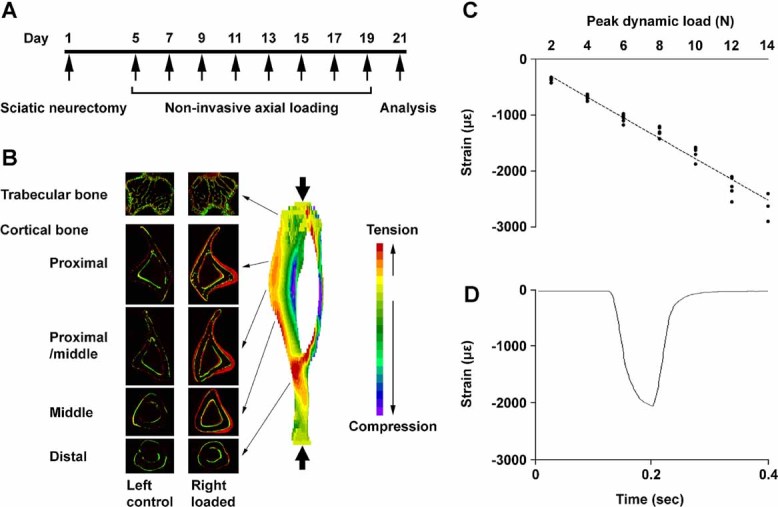
The mouse noninvasive tibia axial loading model. (*A*) Overview of the experimental design. (*B*) Loading-related osteogenesis labeled by calcein green on the first day of loading and alizarin red on the last day of loading (adapted from Ref. 16) and loading-induced strain distribution by finite element analysis (adapted from Ref. 21). (*C*) Relationship between peak dynamic load and strain on the center of the lateral surface in the right proximal/middle tibiae, where predominant osteogenesis can be induced, in 17-week-old mice with right sciatic neurectomy. (*D*) Representative strain recording, induced by a peak dynamic load of 12 N, on the center of the lateral surface in the right proximal/middle tibiae of 17-week-old mice with right sciatic neurectomy.

At 17 weeks of age, a total of 48 mice were divided into eight groups matched for body weight (*n* = 6 per group). Each mouse was subjected to right sciatic neurectomy on day 1 and its right tibia to external mechanical loading under isoflurane-induced anesthesia (see In vivo external mechanical loading below for details) on days 5, 7, 9, 11, 13, 15, 17, and 19 (Fig. 2*A*). Functional adaptation of bone to artificial loading has been previously shown to take place in the tibia when the sciatic nerve is sectioned.^14^, ^15^ Each group received one of the eight graded magnitudes of a dynamic peak load in the right tibia. Except for this difference in peak dynamic load, all groups had the same treatments including the neurectomy and static preload. All mice were able to move in the cages without difficulty and the left tibiae were used as internal controls as validated in the present^16^ and other^17^ loading models. Body weight was measured once a week. At 20 weeks of age (day 21), the mice were euthanized and their lower legs were collected for analysis.

### In vivo external mechanical loading

The apparatus and protocol for noninvasively loading the mouse tibia using a servo hydraulic loading machine and the loading-related osteogenesis by histomorphometry as well as strain distribution by finite element analysis (Fig. 2*B*) has been reported.^16^, ^18^–^21^ In the present study, we first established the apparatus and protocol using an electromagnetic loading machine (ElectroForce 3100; Bose Co., Eden Prairie, MN, USA). In brief, the flexed knee and ankle joints were positioned in concave cups; the upper cup, into which the knee was positioned, was attached to an actuator arm and the lower cup to a dynamic load cell. The tibia was held in place by a 0.5-N continuous static preload for ∼7 minutes, onto which 40 cycles of intermittent dynamic load were superimposed, with a 10-second rest interval between each cycle. One of the eight graded peak dynamic load magnitudes (0, 2, 4, 6, 8, 10, 12, or 14 N) was applied, and the setting of one cycle consisted of (i) 500 N/s to a target peak dynamic load, (ii) a 0.05-second hold at the peak load, and (iii) 500 N/s to the static preload. Forty cycles of load were selected, because this number of cycles is sufficient to stimulate significant loading-related bone gain.^8^ A 10-second rest interval was inserted between cycles because rest interruption enhances the osteogenic effect of loading.^22^, ^23^ Based on the established linear load:strain relationship, strain recordings on the center of the lateral surface in the right proximal/middle tibia, where predominant osteogenesis can be induced by the present axial loading,^16^ in 17-week-old mice with right sciatic neurectomy (Fig. 2*C*, *D*) and strain distribution by finite element analysis^21^ estimated that peak dynamic strain magnitudes on the medial, lateral, and posterior surfaces at the applied loads (0–14 N) ranged from 0 µε (0 N) to approximately 2000, −5000, and −5000 µε (14 N), respectively. The estimated maximum strain rates were approximately 30,000 µε/s on the medial surface and approximately −75,000 µε/s on the lateral and posterior surfaces (500 N/s). The graded peak strain magnitudes included the highest attainable physiological levels while the maximum strain rates were within the levels reported to occur during vigorous activities in animals.^24^

### In vivo mechanical strain measurements

Strain recordings during locomotion were obtained from the right tibiae of mice with right sciatic neurectomy as well as intact mice at 17 weeks of age. In brief, after a subcutaneous injection of buprenorphine hydrochloride (Vetergestic; Reckitt Benckiser Healthcare Ltd., Hull, UK) at 0.1 mg/kg, a trimmed single-element strain gauge (EA-06-015DJ-120; Vishay Measurements Group, Basingstoke, UK) was attached in longitudinal alignment to the center of the medial surface at the proximal/middle site under isoflurane-induced anesthesia, using the previously reported technique.^18^ The medial surface rather than the lateral surface was chosen because the attachment can be made less invasively due to the smaller amount of soft tissue. Strain levels during locomotion were then compared with those engendered by external dynamic loads at the same site.

### High-resolution µCT analysis

Because the mouse bone is small and the present axial loading-related osteogenesis is site-specific,^16^, ^25^ high-resolution µCT was used to quantify three-dimensional bone architecture at precisely comparable sites of the loaded and contralateral control limbs. The lower legs were stored in 70% ethanol and mounted in a plastic tube wrapped in plastic film to prevent drying during scanning. The whole tibiae and surrounding muscles were imaged in the SkyScan 1172 (SkyScan, Kontich, Belgium) with a voxel size of 5 µm^3^. The applied X-ray voltage was 50 kV with 0.5 mm aluminum filtration. The scans were over 180 degrees with a 0.5-degree rotation step. The images were reconstructed and binarized with global thresholding using the SkyScan software. The change ([right – left]/left) in muscle area (Mu.Ar) of lower legs at the level of middle tibia was quantified using the SkyScan software to validate successful sciatic neurectomy-induced disuse.

Morphometric parameters were calculated using the SkyScan software for cortical bone (0.5-mm-long section at 25% [proximal], 37% [proximal/middle], 50% [middle], and 75% [distal] of the bone's longitudinal length from its proximal end) and trabecular bone (secondary spongiosa; 0.25–0.75 mm distal to the growth plate) in the tibiae. In the trabecular region, an irregular, anatomic region of interest adjacent to the endocortical boundary was analyzed. According to the guidelines for assessment of bone microstructure in rodents using µCT,^26^ we evaluated changes ([right – left]/left) in cortical bone area (Ct.Ar), total cross-sectional area inside the periosteal envelope (Tt.Ar), marrow area (Ma.Ar), cortical area fraction (Ct.Ar/Tt.Ar), cortical thickness (Ct.Th), and polar moment of inertia (J), a parameter of structural bone strength, at the four cortical sites, and bone volume fraction (BV/TV), trabecular number (Tb.N), trabecular thickness (Tb.Th), and trabecular separation (Tb.Sp) in the trabecular region.

### Statistical analysis

To assess whether the relationship between peak dynamic load and changes in cortical and trabecular bone mass/strength (Ct.Ar, J, and BV/TV) includes an unresponsive “lazy zone,” a piecewise linear multilevel regression model was fitted. This model consisted of three segments with the middle segment constrained to have value zero and no change in these bone variables with a change in peak dynamic load. The locations of the two change points of peak dynamic load between segments were chosen by a grid search method where all values from 2.5 N to 13.5 N (in steps of 0.1 N) were considered for each change point and the pair of change points that minimized the model deviance selected. If the two change points had the same value, then effectively this would imply a two-stage model, indicating a change of slope but no “lazy zone.” On the basis of our hypothesis, we also performed linear and quadratic multilevel regression analyses on peak dynamic load for all 28 bone variables. Multilevel modeling was used to account for cage/group effects in the analysis. The 95% confidence intervals for minimum effective load, the threshold of peak dynamic load for a decrease or an increase, and the *p* values about comparisons between the proximal/middle site and other three cortical sites were calculated via Monte Carlo simulation. Body weight and longitudinal lengths of left and right tibiae among eight groups were compared by one-way ANOVA. A change in each µCT variable in each group was evaluated by paired *t* tests. The multilevel regression analyses were performed using MLwiN Version 2.10^27^ and other analyses done using GraphPad Prism 5 (GraphPad, Inc., San Diego, CA, USA). A *p* value <0.05 was considered to be significant.

## Results

Among eight graded load groups, the ranges of the mean body weight and longitudinal length of left control tibia were 21.3 to 22.6 g and 17.8 to 18.1 mm, respectively, and the mean differences in the longitudinal lengths of left and right tibiae were within 0.3% at 20 weeks of age. These data indicate that all groups had similar bone size as well as body weight and that the applied dynamic loads did not affect tibia's longitudinal length.

Changes ([right – left]/left) in all µCT variables in each group are shown in Table 1. The minimum change in Mu.Ar was −34.7%, and significant decreases in Ct.Ar and BV/TV were observed in the 0-N group. These data validate that right sciatic neurectomy-induced disuse was successful in all mice.

**Table 1:** Changes ([right – left]/left) in Muscle Area of Lower Legs and Cortical and Trabecular Bone Variables of the Tibias in 20-Week-Old Mice That Received Right Sciatic Neurectomy and Axial Loading in the Right Tibia

	Dynamic load (N)							
	
	0	2	4	6	8	10	12	14
Muscle								
Mu.Ar (%)	−38.8 ± 1.4*	−40.6 ± 1.8*	−40.3 ± 1.5*	−41.2 ± 1.8*	−40.8 ± 0.9*	−40.8 ± 0.7*	−41.9 ± 1.3*	−41.1 ± 0.7*
Cortical bone								
Proximal site								
Ct.Ar (%)	−14.7 ± 1.8**	−17.4 ± 1.7*	−14.3 ± 0.7*	−10.0 ± 2.0**	−0.6 ± 1.2	6.0 ± 2.4	12.6 ± 2.0**	49.4 ± 12.3**
Tt.Ar (%)	−2.4 ± 2.9	−4.4 ± 2.6	−1.3 ± 1.9	−2.1 ± 0.5**	1.9 ± 2.5	2.1 ± 2.4	5.9 ± 2.1***	22.3 ± 6.1***
Ma.Ar (%)	11.3 ± 5.7	8.4 ± 4.5	13.1 ± 4.7***	6.4 ± 2.2***	5.3 ± 5.4	−2.0 ± 3.0	−0.8 ± 3.3	−4.6 ± 4.2
Ct.Ar/Tt.Ar (%)	−12.3 ± 2.8**	−13.4 ± 1.5**	−13.1 ± 1.7**	−8.1 ± 1.9***	−2.2 ± 2.4	4.0 ± 1.4***	6.4 ± 1.6***	21.3 ± 4.9**
Ct.Th (%)	−15.4 ± 1.3*	−14.6 ± 1.1*	−12.5 ± 1.5**	−12.0 ± 2.6**	0.0 ± 0.8	8.3 ± 2.5***	12.1 ± 2.6**	4.6 ± 4.5
J (%)	−10.1 ± 2.8***	−14.0 ± 3.5***	−14.5 ± 2.1**	−10.6 ± 1.9**	0.3 ± 1.7	6.9 ± 5.3	19.9 ± 3.8**	49.3 ± 11.8**
Proximal/middle site								
Ct.Ar (%)	−10.3 ± 1.1*	−10.4 ± 0.5*	−8.4 ± 1.7**	−5.1 ± 2.2	3.0 ± 0.8***	10.1 ± 2.2**	16.2 ± 2.3**	49.0 ± 8.9**
Tt.Ar (%)	2.9 ± 1.8	1.2 ± 2.0	1.3 ± 1.4	1.2 ± 0.7	6.1 ± 0.7*	7.1 ± 2.3***	11.5 ± 2.2**	24.0 ± 4.6**
Ma.Ar (%)	19.9 ± 4.5**	14.8 ± 5.0***	13.4 ± 2.5**	9.2 ± 2.1**	10.2 ± 1.8**	3.4 ± 3.3	5.9 ± 3.1	−4.7 ± 4.0
Ct.Ar/Tt.Ar (%)	−12.7 ± 1.6**	−11.3 ± 2.0**	−9.5 ± 1.3**	−6.2 ± 1.7***	−2.9 ± 0.9***	2.8 ± 1.3	4.2 ± 1.2***	19.8 ± 3.7**
Ct.Th (%)	−14.4 ± 1.7**	−12.6 ± 1.0*	−8.3 ± 1.4**	−5.3 ± 2.6	1.3 ± 2.1	9.8 ± 2.2**	10.4 ± 2.1**	4.8 ± 5.7
J (%)	−5.4 ± 2.5	−7.8 ± 2.1***	−7.3 ± 2.0***	−4.8 ± 2.6	7.1 ± 1.2**	12.5 ± 4.6***	23.6 ± 3.7**	49.2 ± 8.1**
Middle site								
Ct.Ar (%)	−12.5 ± 1.2*	−12.8 ± 1.2*	−7.9 ± 0.7*	−3.6 ± 1.2***	3.1 ± 0.8***	14.4 ± 1.0*	15.5 ± 2.1**	39.5 ± 6.8**
Tt.Ar (%)	−1.1 ± 1.3	−1.6 ± 0.9	−0.8 ± 1.4	−1.4 ± 1.1	2.4 ± 1.7	4.6 ± 0.9**	8.3 ± 1.6**	15.8 ± 3.1**
Ma.Ar (%)	13.0 ± 2.6**	10.4 ± 1.8**	7.5 ± 2.8***	1.5 ± 2.5	1.8 ± 3.7	−6.5 ± 2.2***	0.1 ± 3.6	−12.2 ± 1.9**
Ct.Ar/Tt.Ar (%)	−11.5 ± 1.1*	−11.4 ± 1.0*	−7.1 ± 0.9**	−2.2 ± 1.3	0.9 ± 1.8	9.4 ± 1.2**	6.7 ± 2.2***	20.2 ± 2.8**
Ct.Th (%)	−14.3 ± 1.2*	−13.7 ± 1.2*	−9.1 ± 0.7*	−2.9 ± 1.7	2.3 ± 1.9	14.0 ± 1.8**	11.0 ± 2.6**	8.8 ± 2.6***
J (%)	−10.9 ± 2.3**	−12.1 ± 1.7**	−6.5 ± 2.4***	−4.6 ± 2.0	4.9 ± 2.7	15.9 ± 1.8*	24.3 ± 3.1*	49.4 ± 8.8**
Distal site								
Ct.Ar (%)	−8.2 ± 1.3**	−9.1 ± 1.0*	−6.7 ± 1.2**	−5.1 ± 2.4	−2.1 ± 1.0	0.4 ± 1.6	4.3 ± 1.3***	7.9 ± 2.3***
Tt.Ar (%)	−0.1 ± 0.5	−2.0 ± 0.8	0.6 ± 1.0	−0.2 ± 1.2	−0.5 ± 0.4	1.7 ± 1.5	4.1 ± 0.9**	4.7 ± 2.6
Ma.Ar (%)	15.7 ± 3.2**	9.3 ± 1.7**	13.2 ± 1.5*	9.1 ± 2.7***	2.8 ± 2.5	4.2 ± 3.2	3.6 ± 3.0	−1.3 ± 3.4
Ct.Ar/Tt.Ar (%)	−8.1 ± 1.3**	−7.2 ± 0.8*	−7.2 ± 0.5*	−5.0 ± 1.6***	−1.6 ± 1.2	−1.3 ± 1.3	0.3 ± 1.3	3.1 ± 0.7**
Ct.Th (%)	−10.6 ± 1.1*	−10.4 ± 1.2*	−9.1 ± 0.8*	−5.4 ± 1.8***	−2.6 ± 1.5	−4.4 ± 2.5	0.5 ± 1.8	1.6 ± 2.9
J (%)	−4.5 ± 1.3***	−7.9 ± 1.5**	−3.3 ± 2.1	−3.2 ± 2.9	−2.4 ± 0.4**	1.5 ± 2.5	7.4 ± 1.5**	10.3 ± 4.6
Trabecular bone								
BV/TV (%)	−28.8 ± 2.9**	−34.0 ± 2.7*	−24.8 ± 3.4*	−5.4 ± 1.9	4.8 ± 3.0	42.2 ± 4.6*	30.2 ± 4.5**	87.2 ± 9.3*
Tb.N (%)	−13.3 ± 2.6**	−25.1 ± 2.6**	−15.3 ± 3.3**	−2.6 ± 2.1	4.9 ± 2.1***	21.2 ± 2.2*	12.6 ± 4.5	36.7 ± 6.9**
Tb.Th (%)	−17.9 ± 1.8*	−12.0 ± 1.2*	−11.2 ± 2.2**	−2.7 ± 1.2	−0.1 ± 1.9	17.2 ± 2.4**	15.8 ± 2.7**	37.1 ± 3.2*
Tb.Sp (%)	−1.3 ± 1.9	5.1 ± 3.3	3.4 ± 1.6	−0.6 ± 0.7	−2.0 ± 2.7	−4.4 ± 0.7**	−0.5 ± 3.5	−3.4 ± 2.8

Mean ± SE (*n* = 6 in each).

Mu.Ar = muscle area; Ct.Ar = cortical bone area; Tt.Ar = total cross-sectional area inside the periosteal envelope; Ma.Ar = marrow area; Ct.Ar/Tt.Ar = cortical area fraction; Ct.Th = cortical thickness; J = polar moment of inertia; BV/TV = bone volume fraction; Tb.N = trabecular number; Tb.Th = trabecular thickness; Tb.Sp = trabecular separation.

* *p* < 0.001,

** *p* < 0.01,

*** *p* < 0.05 by paired *t* tests (left versus right).

**Fig. 3:**
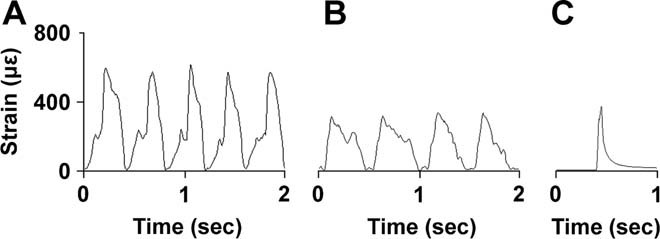
Strain levels in vivo on the center of the medial surface in the right proximal/middle tibiae. (*A*) Representative recording during walking activity in 17-week-old intact mice. (*B*) Representative recording during walking activity in 17-week-old mice with right sciatic neurectomy. (*C*) Representative recording induced by a peak dynamic load of 2 N in 17-week-old mice with right sciatic neurectomy.

Strain measurements during walking activity confirm that in vivo levels of peak strain on the center of the medial surface in the right proximal/middle tibia were lower in mice with right sciatic neurectomy (∼300 µε) than in intact mice (∼600 µε) (Fig. 3*A*, *B*). When the internal and external levels of peak strain were compared in the right tibiae of mice with right sciatic neurectomy, a peak dynamic load of 2-N resulted in a similar level of peak strain during walking activity (Fig. 3*B*, *C*). Consistent with this finding, the values of Ct.Ar, J, and BV/TV between the 0-N and 2-N groups were similar (Table 1).

**Fig. 4:**
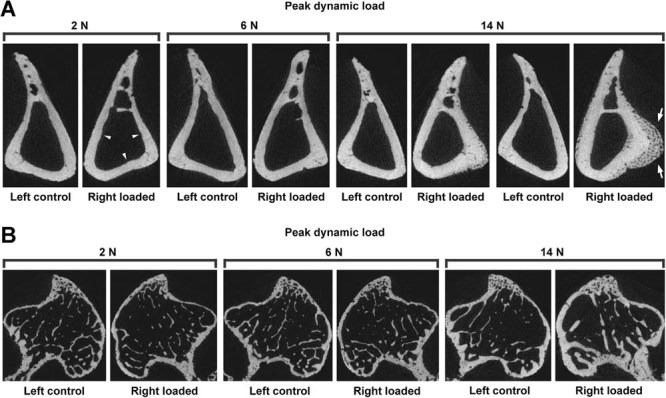
Representative transverse µCT images of the left and right tibiae in 20-week-old mice that received right sciatic neurectomy and axial loading in the right tibia. (*A*) Cortical images at the proximal/middle site. No significant change is observed in the 6-N group. In contrast, in the right side, the 2-N and 14-N groups show bone loss and gain, respectively. Note that, in one-half of the 14-N (highest load) group, loading-related apparent woven bone formation is evident on the lateral and posterior surfaces. (*B*) Trabecular images in the secondary spongiosa. No significant change is observed in the 6-N group. In contrast, in the right side, the 2-N and 14-N groups show bone loss and gain, respectively.

As shown in the µCT images (Fig. 4), in the 14-N (highest load) group, but not any other group, woven bone formation was observed on the lateral and posterior surfaces of the proximal to middle cortical site in one-half of mice (*n* = 3). Compared to the remaining mice without apparent woven bone formation (*n* = 3) in the 14-N group, the loading-related changes in bone mass of these mice were markedly higher not only in the cortical region (Ct.Ar: 23.2% ± 4.8% versus 75.5% ± 6.8% at the proximal site, 30.1% ± 4.3% versus 67.9% ± 5.0% at the proximal/middle site, 24.7% ± 2.2% versus 54.3% ± 2.5% at the middle site, and 4.4% ± 1.0% versus 11.4% ± 3.7% at the distal site) but also in the trabecular region where no woven bone formation was observed (BV/TV: 70.8% ± 8.6% versus 103.6% ± 9.4%).

To assess the physiological relationship between peak dynamic load and bone variables, a total of 9 mice (6 mice in the 0-N group and 3 mice with apparent woven bone formation in the 14-N group) were excluded from the regression analyses. The grid search for the piecewise linear multilevel regression analysis of Ct.Ar, J, and BV/TV found that the two change points were all coincident and thus a two-stage model was being fit, indicating continuously positive relationships between peak dynamic load and changes in cortical and trabecular bone mass/strength that did not include an unresponsive “lazy zone.” In comparison between the linear and quadratic multilevel regression analyses of the 28 bone variables, a quadratic relationship fitted better for nine variables but the improvements in fit compared to a linear relationship were all minor. We therefore present here the results obtained by the linear multilevel regression analysis.

**Fig. 5:**
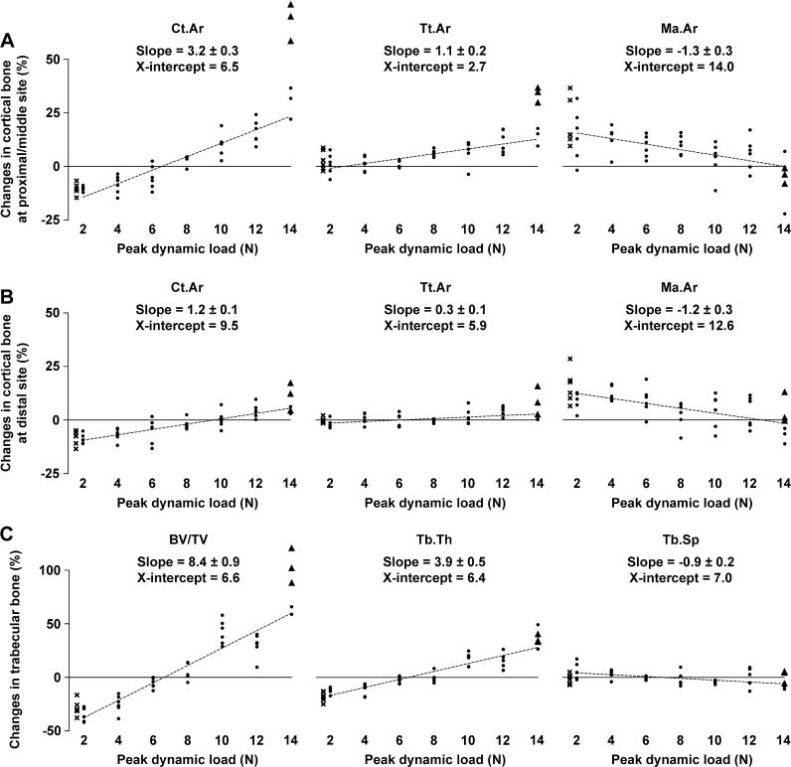
Relationship between peak dynamic load and the changes ([right – left]/left) in bone variables in the tibiae of 20-week-old mice that received right sciatic neurectomy and axial loading in the right tibia. (*A*) Cortical bone area (Ct.Ar), total cross-sectional area inside the periosteal envelope (Tt.Ar), and marrow area (Ma.Ar) at the proximal/middle site. (*B*) Ct.Ar, Tt.Ar, and Ma.Ar at the distal site. (*C*) Bone volume fraction (BV/TV), trabecular thickness (Tb.Th), and trabecular separation (Tb.Sp) in the secondary spongiosa. Best-fit and SE values of slope and best-fit values of x-intercept are shown. • = mice with no apparent woven bone formation; × = mice without external dynamic loading; ▴ = mice with apparent woven bone formation. Note that × (*n* = 6) and ▴ (*n* = 3) were excluded for the multilevel regression analyses.

**Fig. 6:**
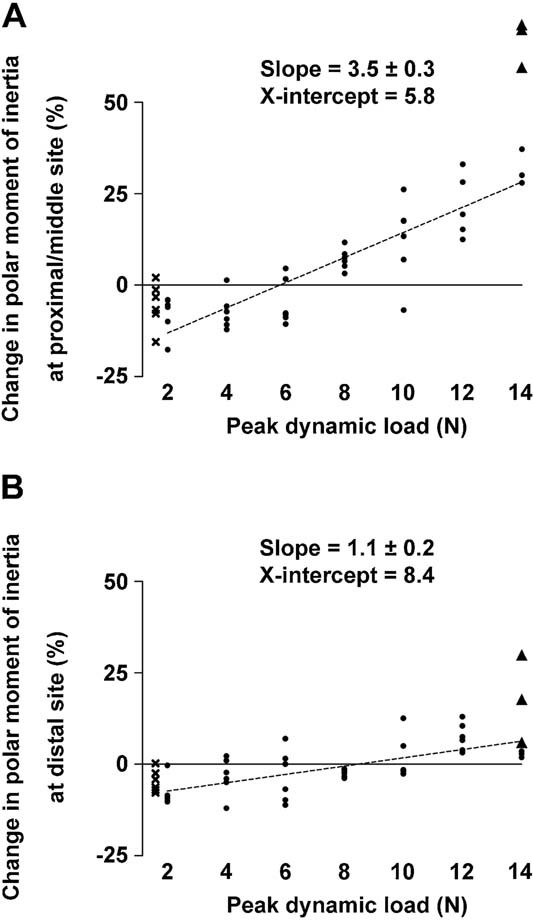
Relationship between peak dynamic load and the change ([right – left]/left) in polar moment of inertia (J), a parameter of structural bone strength, in the tibiae of 20-week-old mice that received right sciatic neurectomy and axial loading in the right tibia. (*A*) Proximal/middle site. (*B*) Distal site. Best-fit and SE values of slope and best-fit values of x-intercept are shown. • = mice with no apparent woven bone formation; × = mice without external dynamic loading; ▴ = mice with apparent woven bone formation. Note that × (*n* = 6) and ▴ (*n* = 3) were excluded for the multilevel regression analyses.

In the cortical regions, the change in Ct.Ar increased with increasing peak dynamic load at all four sites (Table 1; Fig. 5*A*, *B*). This was linked to a positive relationship with Tt.Ar and an inverse relationship with Ma.Ar. The relation with peak dynamic load was similar between Ct.Ar and J, a parameter of structural bone strength (Fig. 6). As summarized in Table 2, there was site-specific difference in minimum effective load, the threshold of peak dynamic load for a decrease or an increase, in cortical variables. Specifically, minimum effective loads of most cortical variables were significantly higher at the distal site compared to the proximal/middle site, though the minimum effective load of Ma.Ar was similar between these two sites (Fig. 5*A*, *B*). The load:response relationship was also site-specific; best-fit ± SE values (% change per N) in Ct.Ar and J were 3.3 ± 0.2 and 3.5 ± 0.4 at the proximal site, 3.2 ± 0.3 and 3.5 ± 0.3 at the proximal/middle site, 3.1 ± 0.2 and 3.7 ± 0.3 at the middle site, and 1.2 ± 0.1 and 1.1 ± 0.2 at the distal site, respectively.

**Table 2:** Minimum Effective Load in Cortical Variables of the Tibias in 20-Week-Old Mice That Received Right Sciatic Neurectomy and Axial Loading in the Right Tibia by Linear Multilevel Regression Analysis

	Ct.Ar	Tt.Ar	Ma.Ar	Ct.Ar/Tt.Ar	Ct.Th	J
Proximal site						
Best-fit value (N)	8.1	6.6	10.5	8.9	8.3	7.6
95% confidence interval (N)	7.7–8.6*	4.7–8.0**	8.3–15.4	8.2–9.6	7.4–9.2***	6.7–8.3*
Proximal/middle site						
Best-fit value (N)	6.5	2.7	14.0	8.8	7.3	5.8
95% confidence interval (N)	5.8–7.2	0.3–4.2	11.3–20.9	8.1–9.6	6.7–7.9	4.9–6.6
Middle site						
Best-fit value (N)	6.5	5.1	8.6	7.3	7.1	6.0
95% confidence interval (N)	6.0–7.0	3.7–6.1***	7.1–10.6**	6.5–8.0*	6.2–7.9	5.4–6.5
Distal site						
Best-fit value (N)	9.5	5.9	12.6	10.9	11.2	8.4
95% confidence interval (N)	8.6–10.6*	0.2–8.8	10.4–17.4	9.8–12.5*	10.0–13.2*	7.1–9.9*

Ct.Ar = cortical bone area; Tt.Ar = total cross-sectional area inside the periosteal envelope; Ma.Ar = marrow area; Ct.Ar/Tt.Ar = cortical area fraction; Ct.Th = cortical thickness; J = polar moment of inertia.

* *p* < 0.001,

** *p* < 0.01,

*** *p* < 0.05 versus proximal/middle site.

Similarly, in the trabecular region, the change in BV/TV increased with increasing peak dynamic load (Table 1; Fig. 5*C*). This resulted primarily from a positive relationship with Tb.Th and also with an inverse relationship with Tb.Sp. Minimum effective loads in trabecular variables are shown in Table 3. The load:response relationship for measures of bone mass was markedly higher in the trabecular region than in the cortical regions; best-fit ± SE value (% change per N) in BV/TV was 8.4 ± 0.9.

**Table 3:** Minimum Effective Load in Trabecular Variables of the Proximal Tibias in 20-Week-Old Mice That Received Right Sciatic Neurectomy and Axial Loading in the Right Tibia by Linear Multilevel Regression Analysis

	BV/TV	Tb.N	Tb.Th	Tb.Sp
Best-fit value (N)	6.6	7.3	6.4	7.0
95% confidence interval (N)	5.7–7.4	6.2–8.3	5.3–7.4	4.3–9.3

BV/TV = bone volume fraction; Tb.N = trabecular number; Tb.Th = trabecular thickness; Tb.Sp = trabecular separation.

Regarding possible cross-talk between bone and adjacent muscle, there was no indication of any relationship between the changes in the mass of bone induced by graded loads and those of its adjacent muscle (Table 1).

## Discussion

The data presented here show a continuous, progressive, and essentially linear relationship between measures of bone mass/strength derived from high-resolution µCT and the peak dynamic loads applied to the mouse tibia across the range, from those sufficient to engender the low strains associated with disuse, through the normal locomotor strain range, to the high strains that in one-half of the animals engendered a change in the character of new bone deposited from lamellar to woven bone. This linearity of response conflicts with the idea that there is a zone of nonresponsiveness surrounding normal peak functional strain levels and suggests that the apparently unresponsive zone seen in experimental situations where artificial and natural loading coexist^10^ is a reflection of the lack of need for adaptation when artificial loading does not produce an osteogenic stimulus greater than that from natural loading. The slope of the strain:response relationship and the minimum effective strain (MES), both of which are determined by features of the strain waveform including peak strain,^6^ strain rate,^7^ and frequency,^28^ are site-specific possibly due to differences in the mismatch in strain distribution^8^ at the different locations between that engendered by artificial loading and that from their natural loading to which they are adapted.

One-half of the highest (14-N) load group showed woven bone formation on the lateral and posterior periosteal surfaces of the proximal/middle tibiae where the highest level of strain is induced (peak strain magnitude: ∼5000 µε, maximum strain rate: ∼75,000 µε/s). This difference in the character of the new bone deposited, which resulted in the measured gain in bone mass being greatly increased, marks the higher limit of the linear response region between strain and new bone formation. In our view, the transition from lamellar to woven bone formation indicates a transition in the rate at which new bone is required. While this transition point is frequently exceeded in pathological conditions, we do not consider that the stimulus need be essentially pathological.^29^, ^30^

The data in our present study are entirely compatible with those from the functionally isolated avian ulna^6^ and from Turner and colleagues^10^ and Sawakami and colleagues,^31^ who applied external loads to the rat tibia and the mouse ulna, respectively. For experiments in which artificial and natural loading coexist,^10^, ^31^ artificial loading will stimulate an increase in bone mass/strength only when this stimulus exceeds that already derived from natural loading. The results published by Turner and colleagues^10^ and Sawakami and colleagues^31^ show this threshold on the endocortical surface of the rat tibia and the mouse ulna, respectively (Fig. 1*B*). The threshold is also a feature of our own experience in the tibiae of intact 17-week-old female non-neurectomized C57BL/6 mice. To examine the bone's adaptive response to loading across the complete strain range, we had hoped to construct an experimental situation of zero strain. However, we could not do this, because even in sciatic neurectomized limbs locomotion engendered strains similar in magnitude to those resulting from a peak dynamic load of 2 N (Fig. 3*B*, *C*). Predictably, this level of external loading produced no change in bone mass/strength. A similar situation can be seen in the surgically isolated avian ulna^6^ where a peak dynamic strain of 500 µε engendered by external loading had no effect on cortical bone area (Fig. 1*C*).

Coincidence in the MES values between the avian ulna (∼1000 µε on the periosteal surface at the middle site) in Rubin and Lanyon's earlier study,^6^ the rat tibia (∼1000 µε on endocortical surface at the middle site) in Turner and colleagues report,^10^ and the mouse tibia (6.5 N = ∼1000 µε on the center of the lateral periosteal surface at the proximal/middle site) in our current experiment should not be taken as indicating that this is a universal value, as shown by the significant difference in response between the proximal/middle and distal cortical sites where loading-induced peak strains, determined by finite element analysis, are similar.^21^ The strain-related stimulus includes peak strain,^6^ strain rate,^7^ strain distribution,^8^ and thus strain gradients.^32^ It is difficult to assess the extent to which the location dependency is due to variations in the mismatch between the natural and artificial strain distributions^8^ and strain gradients.^32^ However, such variation is a probable explanation for why a peak dynamic load of 8 N that stimulated adaptive change in the bone's proximal/middle portion produced no effect in the distal portion (Fig. 5*A*, *B*). A similar difference in regional response to a single loading regimen within a bone has been reported in the rat ulna.^33^

Acceptance of a linear osteoregulatory response to strain between near zero and the lamellar/woven bone transition has some experimental advantages because it allows the slope of the strain:response and the MES to be compared in animals of different phenotype using only a single loading and sciatic neurectomy test group in each population. Revealing genetic differences in MES^31^, ^34^ may provide information on the mechanisms involved in regulation of bone mass. Lowering the MES pharmacologically^25^, ^35^ is an attractive strategy for the prevention of fragility fractures.

**Fig. 7:**
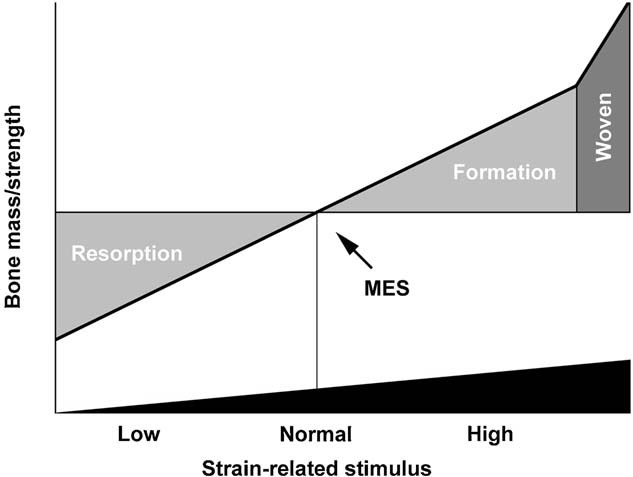
A schematic diagram illustrating the progressive, essentially linear, increase in bone mass/strength with increasing strain-related stimulus derived from functional load-bearing. In a bone that has already adapted to any level of load-bearing, any increase or decrease in strain-related stimulus will be associated with an increase or a decrease, respectively, in bone mass/strength. At one extreme, bone loss will continue until a genetically determined minimum level is achieved. At the other extreme, the osteogenic response to loading will involve exuberant woven bone formation. This level of strain will probably be associated with increased levels of microdamage. MES = minimum effective strain.

In conclusion, multilevel regression analyses of three-dimensional bone architecture in the left control and right loaded tibiae of skeletally mature mice with right sciatic neurectomy shows that the relationships between peak dynamic load and measures of bone mass/strength in both cortical and trabecular regions are essentially linear between the low strains associated with disuse (∼300 µε) and the high strains associated with the transition from lamellar to woven new bone formation (∼5000 µε). These findings indicate that loading-related functional adaptation of bone mass/strength consists of a continuous, progressive, essentially linear response to the bone's physiological loading with no zone around which the response is different or absent (Fig. 7). This suggests that normality of bone mass/strength is not achievement of set-points with a surrounding zone of nonresponsiveness but the individually variable result of a continuous stimulus arising from functional adaptation to load-bearing. Normality of bone architecture at each location is thus a reflection of past and present normality of bone loading.
